# Impact of Organic Loading Rate in Volatile Fatty Acids Production and Population Dynamics Using Microalgae Biomass as Substrate

**DOI:** 10.1038/s41598-019-54914-4

**Published:** 2019-12-05

**Authors:** Jose Antonio Magdalena, Silvia Greses, Cristina González-Fernández

**Affiliations:** Biotechnological Processes Unit, IMDEA Energy, Madrid, Spain

**Keywords:** Population dynamics, Environmental biotechnology

## Abstract

Volatile fatty acids (VFAs) are regarded as building blocks with a wide range of applications, including biofuel production. The traditional anaerobic digestion used for biogas production can be alternatively employed for VFAs production. The present study aimed at maximizing VFAs productions from *Chlorella vulgaris* through anaerobic digestion by assessing the effect of stepwise organic loading rates (OLR) increases (3, 6, 9, 12 and 15 g COD L^−1^ d^−1^). The biological system was proven to be robust as organic matter conversion efficiency into VFAs increased from 0.30 ± 0.02 COD-VFAs/COD_in_ at 3 g COD L^−1^ d^−1^ to 0.37 ± 0.02 COD-VFAs/COD_in_ at 12 g COD L^−1^d^−1^. Even though, the hydrolytic step was similar for all studied scenario sCOD/tCOD = 0.52–0.58), the highest OLR (15 g COD L^−1^ d^−1^) did not show any further increase in VFAs conversion (0.29 ± 0.01 COD-VFAs/COD_in_). This fact suggested acidogenesis inhibition at 15 g COD L^−1^d^−1^. Butyric (23–32%), acetic (19–26%) and propionic acids (11–17%) were the most abundant bioproducts. Population dynamics analysis revealed microbial specialization, with a high presence of Firmicutes followed by Bacteroidetes. In addition, this investigation showed the microbial adaptation of Euryarchaeota species at the highest OLR (15 g COD L^−1^d^−1^), evidencing one of the main challenges in VFAs production (out-competition of archaea community to avoid product consumption). Stepwise OLR increase can be regarded as a tool to promote VFAs productions. However, acidogenic inhibition was reported at the highest OLR instead of the traditional hydrolytic barriers. The operational conditions imposed together with the high VFAs and ammonium concentrations might have affected the system yields. The relative abundance of Firmicutes (74%) and Bacteroidetes (20%), as main phyla, together with the reduction of Euryarchaeota phylum (0.5%) were found the best combination to promote organic matter conversion into VFAs.

## Introduction

Volatile fatty acids (VFAs) are valuable chemicals produced during the middle stages (acidogenesis and acetogenesis) of anaerobic digestion (AD)^1^. The interest in VFAs relies on their use as building blocks within the renewable-based biorefinery concept^2–4^. AD is a complex organic matter degradation process composed of four different phases (hydrolysis, acidogenesis, acetogenesis and methanogenesis). Numerous reactions and microorganisms interact to transform the organic matter firstly into intermediate products (VFAs) and finally into biogas. AD optimization towards VFAs accumulation need to circumvent VFAs consumption in the methanogenic stage^5^. In this sense, different strategies have been adopted to drive AD to VFAs production such as the use of specific substrates, manipulation of operational conditions or the use of microbial biomass rich in organic acids producers^6,7^.

With regard to the substrate, the use of microalgae biomass presents potential advantages for the process because of the high protein content exhibited by some strains. During AD, proteins degradation results in the release of ammonium and free ammonia to the medium, which could cause the destabilization of the AD process. As a matter of fact, high concentration of these compounds is toxic for methanogenic archaea, which in turn promotes VFAs accumulation^8,9^.

Manipulation of operational conditions such as pH, temperature, hydraulic retention time (HRT) and organic loading rate (OLR) must be taken into account when targeting VFAs production^10–12^. OLR expresses the amount of organic matter fed into a system in terms of Chemical Oxygen Demand (COD). High OLR values can lead to pH drop due to the fast generation of VFAs. Indeed, changes in OLR affect AD process in terms of population dynamics and organic matter availability and thus, final VFAs productions yields and profile might also vary. VFAs can be obtained from a wide amount of substrates^7^. Nevertheless, valorisation of microalgae biomass is an important hotspot due to the key role of this biomass in studies related to wastewater treatment^13^. The novelty of this investigation lies on the use of this feedstock, since the surplus of biomass generated during wastewater bioremediation might constitute an attractive substrate to obtain added-value bio-based compounds. In this context, the study of different OLRs to assess VFAs production from microalgae is relevant to identify system boundaries that should not be overcome to ensure maximum conversion efficiency. In this manner, this study aimed at elucidating the effect of increasing OLR values (3, 6, 9, 12 and 15 g COD L^−1^ d ^−1^) on VFAs production yields and profiles in a continuous stirred tank reactor (CSTR) using *Chlorella vulgaris* (protein rich substrate). Moreover, population dynamics analysis throughout the different scenarios was assessed to find out the involved microorganisms to identify those who develop a key role in VFAs production.

## Methods

### Inoculum and substrate pretreatment

Adapted anaerobic sludge to temperature and substrate was collected from a previous anaerobic reactor set at psychrophilic range temperature (25 °C) and fed with enzymatic pretreated *C. vulgaris*. In this sense, the anaerobic inoculum was adapted to low temperature operation and to the substrate as well. The substrate *C. vulgaris* was purchased from Allmicroalgae (Portugal) revealing a composition (dry weight) of 57.9% proteins (w/w), 21.6% carbohydrates, 13.4% lipids and 7.1% ashes. Since the goal of this study was to investigate the acidogenesis stage, biomass pretreatment was applied to avoid hydrolysis limitation. Commercial enzymatic cocktail “Alcalase 2.5 L” (Novozyme, Denmark) was employed to pretreat the biomass and make available the organic matter to anaerobic microorganisms. The dosage (0.585 UA g^−1^ TS^−1^) and procedure was based on results obtained for *C. vulgaris*^9,14^.

### Experimental set up

AD was carried out under semi-continuous feeding mode in 1 L CSTR. Agitation was performed by mechanical stirring at 250 rpm. The operational temperature was maintained at 25 °C using a water bath and the HRT was set at 8 days^−1^. OLRs applied to test the influence of this parameter on VFAs production allowed dividing the experimental period into five different scenarios (Sc. I-V), as it can be seen in Table 1. pH was monitored but not controlled. Total and soluble COD and N-NH_4_^+^ were measured using test kits (Merck, ISO 15705, ISO 000683). Total and soluble COD together with ammonium, VFAs and pH were measured twice per week. VFAs were measured by liquid chromatography (HPLC) and analysed through an Agilent 1260 HPLC-RID (Agilent) equipped with a Cation H Refill Cartridge Microguard column (Biorad) and an Aminex HPX-87H ion exclusion column (300 × 7.8 mm I.D.) (Biorad). Na^+^ was measured by ion chromatography (ICS 3000, Dionex) equipped with pre-columns and separation columns CG 16 and CS16 (3 mm ø) for cations. The column temperature was set at 35 °C. Biogas composition was analysed by gas chromatography coupled with a thermal conductivity detector (Clarus 580 GC, PerkinElmer) and equipped with an HSN6–60/80 Sulfinert P packed column (7′ ×1/8″ O.D.) and a MS13X4-09SF2 40/60 P packed column (9′ × 1/8″ O.D.) (PerkinElmer). The biological process was considered at steady state condition when VFAs resulted in a constant value and the reactor was operated during 3-HRTs. COD removal was calculated according to Eq. 1, where CODin is the total organic matter fed into the system and COD_out_ is the total organic matter recovered in the effluent:

1 $$ \% {\rm{COD}}\,{\rm{removal}}=\frac{({{\rm{COD}}}_{{\rm{in}}}-{{\rm{COD}}}_{{\rm{out}}})}{{{\rm{COD}}}_{{\rm{in}}}}\cdot 100$$

**Table 1:** Average values achieved throughout the different scenarios of the CSTR operation.

	OLR (g COD·d^−1^·L^−1^)	tCOD (g L^−1^)	% COD removal	sCOD (g L^−1^)	TS (g L^−1^)	VS (g L^−1^)	pH	NH_4_^+^ (g L^−1^)	%CH_4_	%CO_2_	VFAs (g COD L^−1^)	VFAs-COD/COD_in_
Sc. I	3	21.9 ± 3.2	29.3 ± 6.1	11.4 ± 0.9	8.3 ± 0.5	6.3 ± 0.5	6.3 ± 0.3	0.9 ± 0.1	39.5 ± 11.9	60.5 ± 11.9	9.1 ± 0.6	0.30 ± 0.02
Sc. II	6	38.3 ± 0.8	20.1 ± 1.9	20.1 ± 3.7	14.2 ± 1.4	10.2 ± 0.4	6.3 ± 0.1	1.4 ± 0.2	25.7 ± 5.5	68.9 ± 5.6	16.5 ± 3.2	0.34 ± 0.01
Sc. III	9	69.6 ± 1.3	3.3 ± 1.8	29.9 ± 3.2	24.9 ± 0.5	19.6 ± 0.5	6.3 ± 0.1	2.4 ± 0.3	15.4 ± 1.9	79.5 ± 2.4	28.0 ± 2.3	0.39 ± 0.04
Sc. IV	12	91.1 ± 6.2	2.2 ± 3.1	47.2 ± 5.1	33.1 ± 2.5	27 ± 2.0	6.5 ± 0.1	3.8 ± 0.2	14.2 ± 1.4	80.0 ± 2.1	36.8 ± 2.1	0.37 ± 0.02
Sc. V	15	109.9 ± 3.4	14.1 ± 2.7	62.2 ± 2.9	45.4 ± 0.1	35.5 ± 0.6	6.5 ± 0.1	4.4 ± 0.1	11.7 ± 0.9	83.8 ± 2.1	36.4 ± 1.5	0.29 ± 0.01

*tCOD: total chemical oxygen demand; sCOD: soluble chemical oxygen demand; TS: Total solids; VS: Volatile solids;VFAs: Volatile fatty acids.

### DNA extraction

Once each scenario achieved the steady-state, samples were collected and immediately frozen at −20 °C. DNA was extracted using the kit “FastDNA SPIN Kit for Soil” (MP Biomedicals, LCC), according to the protocol provided by the manufacturer. Quality of the DNA extracted was checked using a nanodrop by measuring 260/280 and 260/230 ratios and the amount of DNA extracted (ng/mL). The primers used for the amplification of the 16 S rRNA gene were 341 F and 805 R (F–CCTACGGGNGGCWGCAG and R–GACTACHVGGGTATCTAATCC), which targeted the hypervariable regions V3 and V4 of both bacteria and archaea. Amplicons resulting from PCR were sequenced on a MiSeq Sequencer (Illumina) by Life Sequencing (University of Valencia, Spain) with MiSeq reagent kit v3 (600-cycle), according to the manufacturer’s protocol. Sequence data were processed by using bioinformatics tools. First of all, paired-end reads were merged using the program PEAR^15^. Afterwards, sequence quality was filtered using PRINSEQ and only sequences with a quality score of 30 and minimum lengths of 350 bp were taken into account for further analysis^16^. Primer sequences were removed using Mothur^17^ while chimeric sequences were removed and the resulted sequences were clustered into operational taxonomic units (OTUs) at 97% sequence identity (OTU 0.97). The latter step was performed by USEARCH using the Greengenes database gg_13_8^18^, which is implemented in the Quantitative Insights Into Microbial Ecology (QIIME) 1.9.1 software package^17,19,20^. These sequence data have been submitted to GenBank database in a project (accession number PRJNA529178).

Regarding microbial statistics, diversity was analysed with estimation of Shannon index as well as the number of observed OTUs, providing the microbial evenness and richness of the samples. To determine changes in the microbiome population due to the OLR increase in the system, weighted UniFrac distance matrix was used to elaborate Principal Coordinate Analysis (PCoA). Besides, the effect of the physicochemical parameters on reactor performance was evaluated by Principal Components Analysis (PCA) using PAST^21^, which takes into account the experimental measurements of each stage of the reactor. Additionally, variances in microbial composition between scenarios were evaluated through the ANOSIM statistical analysis with a p-value of 0.05, also using PAST^21^. The ANOSIM statistical analysis was performed with a p-value of 0.05 in order to test for differences in microbial community composition between the scenarios. This statistical test resulted in the R-values matrix shown in Table S1, where values close to 1 indicated a strong dissimilarity between samples and 0 indicated no difference.

## Results and Discussion

### Reactor performance

The present study aimed at maximizing VFAs productions by assessing the effect of stepwise OLR increases. The initial scenario (Sc. I, 3 COD L^−1^d^−1^) evidenced average tCOD and sCOD values of 21.9 ± 3.2 g COD L^−1^ and 11.4 ± 0.9 g COD L^−1^, respectively, during the steady-state, which corresponded to a COD removal of 29.3 ± 6.1% (Table 1). The COD removal attained during this scenario showed that AD process was not working well for biogas production probably due to the low HRT (8 days) and high OLR (3 g COD·L^−1^·d^−1^) values imposed. Conventional AD processes used for maximizing methane production must have a balanced HRT and OLR, since these are key parameters in process optimization^22^. Too short HRT might cause incomplete substrate degradation or microbial population death by starvation whereas low and high OLR values can drive the process either to starvation or to incomplete organic matter degradation due to inhibition by overloading. As a matter of fact, HRT and OLR values usually employed in literature for microalgal biomass degradation via AD for biogas production are very different to the ones employed in the present study and showed higher COD removals. For instance, 51% COD removal took place in an anaerobic digester fed with *C. vulgaris* (1 g COD L^−1^ d^−1^) at 28 days HRT^23^. Since methanogenic inhibition is desired for VFAs production, the selection of low HRT and high OLR values were appropriated for such a goal. Nevertheless, the COD removal was still high and thus, an important carbon fraction was still lost in the biogas stream. For this reason, further OLR increases were applied.

**Figure 1:**
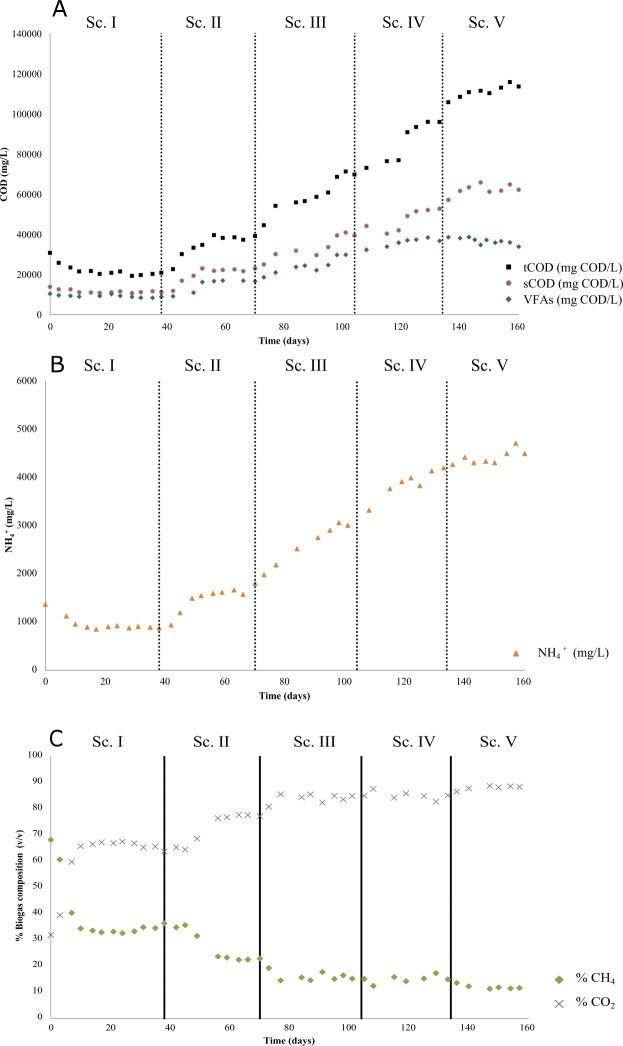
Time course of the main experimental parameters during reactor operation: (**A**) Total/soluble chemical oxygen demand and volatile fatty acids; (**B**) ammonium and (**C**) biogas composition.

Stepwise OLR increases resulted in concomitantly increasing organic matter conversion into VFAs (Fig. 1). Accordingly, Sc. II (6 g COD L^−1^ d^−1^) reached values of 38.3 ± 0.8 and 20.1 ± 3.7 g COD L^−1^ (tCOD and sCOD, respectively, Table 1), reducing the COD removal from 29.3% attained in Sc. I to 20.1%, as a consequence of methanogenic instability. Considerably lower values were achieved at the end of Sc. III-IV (% COD removals less than 5%, tCOD of 69.6 ± 1.3 and 91.1 ± 6.2 g COD L^−1^ and sCOD of 29.9 ± 3.221 and 47.2 ± 5.1 for 9 and 12 g COD L^−1^ d^−1^, respectively). However, when the system was operated at OLR 15 g COD L^−1^ d^−1^ (Sc. V), the COD removal percentage seemed to increase slightly when compared to Sc. IV (14.1 ± 2.7% against <5%). In this latter scenario, an acclimation of the anaerobic archaea community to consecutive OLR increasing values might have taken place, improving the organic matter removal efficiency (Table 1). The adaptive capacity of methanogenic archaea to specific process conditions has been already proven in literature^24,25^. Nevertheless, the recorded values for total COD removal were too low within the carbon balance. As a matter of fact, fermentation of organic compounds by acidogenic bacteria and methanogenic archaea is also devoted for the growth of new cells (0.15 kg VSS/kg COD for acidogenic bacteria and 0.03 kg VSS/kg COD in the case of methane producers)^26^. Overall, the COD removal from Sc. II onwards was considered too low in the carbon flow directed to biogas but percentages were rather attributed to anaerobic microorganism’s growth.

Ammonium (NH_4_^+^) and free ammonia (NH_3_) concentrations are important parameters since high concentrations of these compounds may result inhibitory for methanogenic archaea resulting in methanogenesis inhibition. NH_4_^+^ and NH_3_ accumulation might occur when proteins are degraded during AD^24^. Achieved NH_4_^+^ concentrations showed a growing trend up to 4,410 mg NH_4_^+^-N L^−1^ at 15 g COD L^−1^ d^−1^ (Table 1). Even though NH_4_^+^-N was registered throughout the experimental time, only the two last scenarios (Sc. IV and V 3.8 ± 0.2 and 4.4 ± 0.1 g L^−1^ NH_4_^+^-N, respectively) resulted in values close to the threshold indicated in literature (above 3 g L^−1^ of total ammonia nitrogen) to provoke methanogenesis inhibition^27,28^. With regard to free ammonia (NH_3_), the concentrations attained during the experiment were very low since temperature was set at the psychrophilic range temperature (25 °C) and pH values were always between 6 and 7 (Table 1). According to literature, inhibition due to this compound occurs at 80 mg L^−1^ N-NH_3_^29^. This value was far from the ones attained in the present study (below 10 mg L^−1^ NH_3_-N). Thus, this compound was presumably not the responsible for inhibiting methanogenic archaea but it cannot be neglected that total ammonia (ammonium + ammonia) were in the inhibition level for methanogenic archaea. In this sense, the operational conditions imposed in the system were considered suitable for VFA accumulation rather than consumed by archaea.

### VFAs production: concentration, yields and profiles

**Figure 2:**
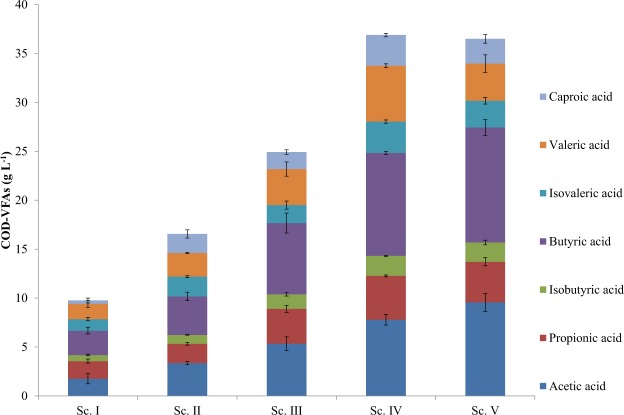
Volatile fatty acids (VFAs) production from scenarios I to V.

An increase in VFAs production (mg COD-VFA L^−1^) was noticed throughout the experimental time at increasing OLR values from Sc. I-V (Fig. 1 and Fig. 2). However, the last scenario fed at 15 g COD L^−1^ d^−1^ resulted in a decrease in VFAs concentration. Similar experiments available in literature conclude on the existence of an optimum OLR value from which VFAs production does not increase. These studies attribute this point of inflection to the hydrolytic capacity of the system. When this point is exceeded, the first step of the AD becomes limiting. For instance, AD of olive mill solid residue was carried out under different OLR values from 3.2 to 15.1 g COD L^−1^ d^−1^ equivalent to HRT from 50 to 10.7 days at continuous feeding mode^30^. Those researchers pointed out that the optimum value was 12.9 g COD L^−1^ d^−1^ (HRT 12.4 days) resulting in VFAs production of 15–20 g COD-VFA L^−1^. A subsequent OLR increase did not report higher VFAs productions. The inhibition of the process was characterized by a strong decrease of the most abundant product acetic acid. VFAs productions were as well monitored in a similar study at OLR 5; 6.6; 10 and 13.3 g COD L^−1^ d^−1^ and decreasing HRT values 4; 3; 2; 1.5 days at mesophilic conditions (37 °C) in a process devoted for biohydrogen production from a waste stream of palm oil^31^. Results showed a maximum VFAs production of 1.5 g VFAs L^−1^ at high OLR values and low HRT (10 g COD L^−1^ d^−1^ and 2 days). Final VFAs productions in these studies were below the ones attained herein, probably due to the use of substrates with different macromolecular composition and operational conditions. Both former studies attributed the drop in VFAs production to a deficient hydrolytic step. At this point, and as mentioned in Section 2.1, the present study subjected microalgae biomass to a proteolytic pretreatment to avoid any hydrolysis limitation with the focus put on the acidogenesis stage of AD. In fact, the ratio sCOD/tCOD comparison of the different scenarios showed quite stable values ranging 0.52–0.58. This fact suggested that the hydrolytic step was not a bottleneck for VFAs production along the increasing OLRs applied since similar ratios were attained (Table 1). Thus, it was inferred that an inhibition of the acidogenic step took place. In this sense, the acidogenic inhibition step has been previously studied and different compounds were pointed out as responsible for the acidogenic inhibition. K^+^, Na^+^, clorophenols and heavy metals (Cu > Zn > Cr > Cd > Ni > Pb) are toxic for acidogenesis^32^. Out of these compounds, sodium may have affected acidogenic activity in the present study, as NaOH was used to control pH during the enzymatic pretreatment of the microalgal biomass. The analysis revealed increasing Na^+^ concentrations from Scenario I to V. This concentration concomitantly increased from 1.02 g L^−1^ determined in Scenario I, 1.8 g L^−1^, 2.8 g L^−1^, 3.7 g L^−1^ and 4.9 g L^−1^ Na^+^ in Scenario V. This compound affects the specific growth rate of microorganisms because it plays a role in the formation of adenosine triphosphate and NADH oxidation. Although it is beneficial at minor concentrations (<1 g L^−1^ Na^+^), higher amounts might alter anaerobic species growth^32^. Since AD has been devoted traditionally for biogas production, the influence of sodium in methanogens has been more studied^33,34^. However, hydrolytic, acidogenic and acetogenic species are known to be more sensitive to Na^+^ ^35^. In this sense, there are studies showing moderate methanogenic inhibition at Na^+^ values ranging 3.5–5.5 g L^−1^ ^36^. Hence, taking into account the acidogenic sensitivity aforementioned, it could be inferred that Na^+^ affected process yields in terms of VFAs production.

Likewise, high ammonium concentrations have been also found to affect the acidogenic step. As a matter of fact, the high ammonium concentrations attained at the highest OLR (4.4 g L^−1^) were above the level (3.1 g L^−1^) identified for acidogenic bacteria inhibition^37^. Finally, high VFAs concentrations have been studied as well as possible inhibitors of the acidogenesis. Investigations found a slight inhibitory effect at 4 g VFAs L^−1^ during the fermentation of glucose^38^. Since these values are far below the VFAs productions obtained in the present study, high VFAs concentrations determined herein could have also hampered the acidogenic stage.

The efficiency of the different scenarios was assessed by calculating the organic matter conversion yields into VFAs (COD-VFAs/COD_in_). Sc. I exhibited the lowest value (0.30 ± 0.02) concomitantly with the highest % COD removal (Table 1). From that point onwards, the system increased organic matter conversion into VFAs in the following scenarios (Sc. II, 0.34 ± 0.01; Sc. III 0.39 ± 0.04; Sc. IV 0.37 ± 0.02 COD-VFAs/COD_in_) until Sc. V, in which conversion dropped (0.29 ± 0.01 COD-VFAs/COD_in_). The lower organic matter conversion efficiency into VFA determined at the highest OLR tested (15 g COD L^−1^d^−1^) was attributed to a combination of high ammonium, VFAs and sodium concentrations. In addition, this decrease in organic matter conversion into VFAs agreed with the higher COD removal registered in Sc. V (Table 1).

VFAs profiles were assessed to evaluate the influence of increasing OLR values (Fig. 2). Butyric acid was the most abundant product obtained in the digesters accounting up to 11.7 ± 0.8 g COD L^−1^ at 15 g COD L^−1^ d^−1^, which corresponded to 32.2% of total VFAs production. This VFA registered an increasing trend from Sc. I to Sc V (25.8% to 32.2%). Accumulation of butyric acid is regarded as a signal of higher hydrogen partial pressure than when the process is devoted to biogas production. In this sense, when hydrogen-utilising methanogens are exposed to hydrogen partial pressures above 10^−4^ atm, VFAs such as butyric acid accumulate in the system^39^. The second most abundant product in each stage was acetic acid (26% in Sc. V out of total VFAs production vs 19–20% in the rest of the stages). This fact might be explained because of the degradation of the longest VFAs (such as isovaleric valeric and caproic acids) into butyric and acetic acids (from 8.6%, 15.4%, and 8.4% isovaleric, valeric and caproic acids, respectively in Sc. IV to 7.5%, 10.4% and 6.9% in Sc. V). Similarly to the present study, another investigation using a mixture of *C. vulgaris* and *Scenedesmus quadricula* as substrate was carried out for VFAs production. Acetic, propionic and butyric acids were also the most abundant products although their distribution was slightly different (46% acetic acid; 19% propionic acid and 19% butyric acid).The latter experiment was carried out in batch mode, which makes difficult the comparison. The underlying abundance or shortage of a concrete VFA is due not only to the substrate employed, but also to the operational conditions set in the system^40^. In addition, different substrates also result in varying VFAs productions. For instance, VFAs production from sucrose for hydrogen production from AD reported a different VFAs profile consisting of 52% butyric acid, 9% propionic acid and 32% acetic acid at 40 g COD L^−1^ d^−1^ and HRT 12 h^41^. Overall, comparison with literature is hard since substrate, feeding mode or operational conditions affect VFA production yields and profiles.

### Microbial population dynamics

#### Microbial community analysis

**Figure 3:**
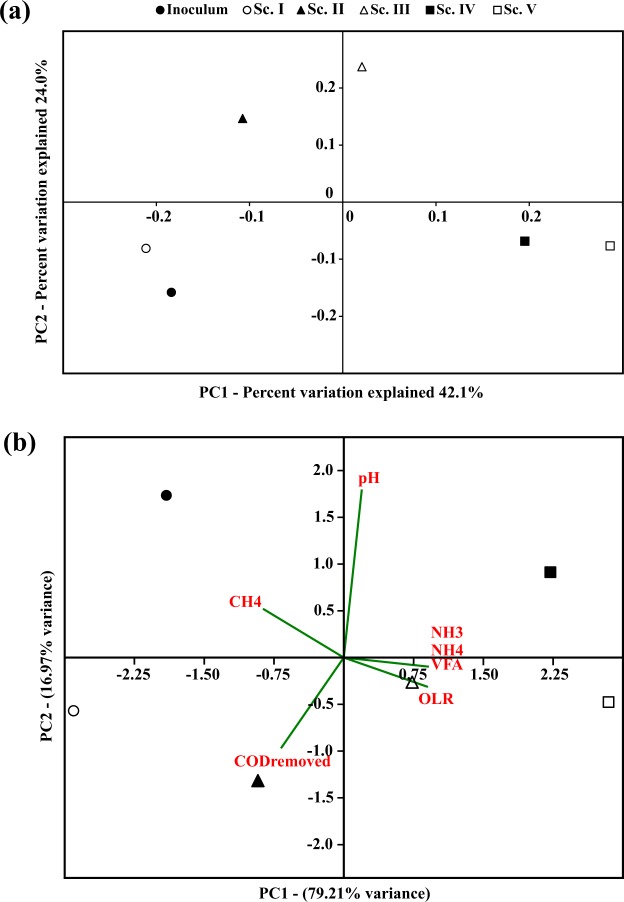
Principal coordinate analysis (PCoA) (**a**) and principal components analysis (PCA) (**b**).

Since promoting specific acidogenic bacteria population is a key factor for maximizing VFA production, microbial communities were analysed during the steady-state of each scenario in order to evaluate the effect of increasing OLR on the relative abundance of the dominant microorganisms. In fact, there was a clear microbial trend along the experimental scenarios in terms of diversity, statistics and microbial distribution analyses. As it can be seen in Table 2, Shannon index reflected a slight diversity increase from the inoculum (3.357) to Sc. I (4.110). During this first scenario, operation of the reactor likely promoted the growth of microorganisms. Likewise, once OLR was increased in the following scenarios (II and III), an increase in Shannon index was detected (4.417 and 4.469, respectively) suggesting an adaptation of the anaerobic biomass present in the reactor to the conditions imposed in the system. However, the subsequent OLR increase in Sc. IV and Sc. V resulted in lower diversity than the previous scenarios (3.870 and 3.802, respectively). These values indicated the specialization of the microorganisms present in the reactor at high OLR values and displayed the crucial influence that this parameter wields over the process. Regarding the number of OTUs observed per sample, these values did not exhibit the same trend that Shannon index (Table 2). It should be taken into account that diversity is not only represented by richness but also by evenness and thus, the higher the microorganisms detected as well as their homogeneity (in terms of relative abundance), the higher the diversity in the system^42^. However, it can be seen that the observed OTUs decreased substantially from Sc. II (153, 200) to Sc. IV (100,400). This fact confirmed the microbial adaptation to the operational conditions and the microbial consortia specialization. The influence of OLR was displayed in the PCoA statistical analysis (Fig. 3a), which reflected that microbial samples were clustered distinctly according to the different OLR ranges: (i) inoculum and Sc. I, (ii) Sc. II-III and (iii) Sc. IV-V. Thus, physico-chemical parameters values changed (N-NH_4_^+^/N-NH_3_, VFAs) due to the progressive OLR increase, definitely affecting microbial populations. pH remained stable along the experimental time. In this sense, the pretreated microalgae fed at pH 8 might have buffered the system, avoiding the pH drop associated normally to high VFAs concentration. As it can be seen in PCA analysis, the VFA concentration registered at the highest OLR was mainly related to the high NH_4_^+^ concentrations released to the medium (Fig. 3b). Both, VFAs and NH_4_^+^, are compounds that might be toxic for the microbiome, explaining the specialization at increasing OLRs^32,37^. An ANOSIM (Fig. S1) test confirmed the strong dissimilarity between the clusters detected through PCoA as well as a high similarity between the scenarios that constituted each cluster. In addition, microorganism’s population changed throughout the different scenarios with a concomitant increase in organic matter conversion into VFAs. However, population changes was not reflected in VFA profiles obtained, which remained stable throughout the different scenarios (Fig. 2).

**Table 2:** OTUs and Shannon and Simpson indices calculated for the samples.

Scenario	Observed OTUs	Shannon
Inoculum	123,000	3.357
I	148,700	4.110
II	213,200	4.447
III	178,600	4.469
IV	166,600	3.809
V	144,400	3.889

OTUs: Operational taxonomic units.

#### Microbial community composition

**Figure 4:**
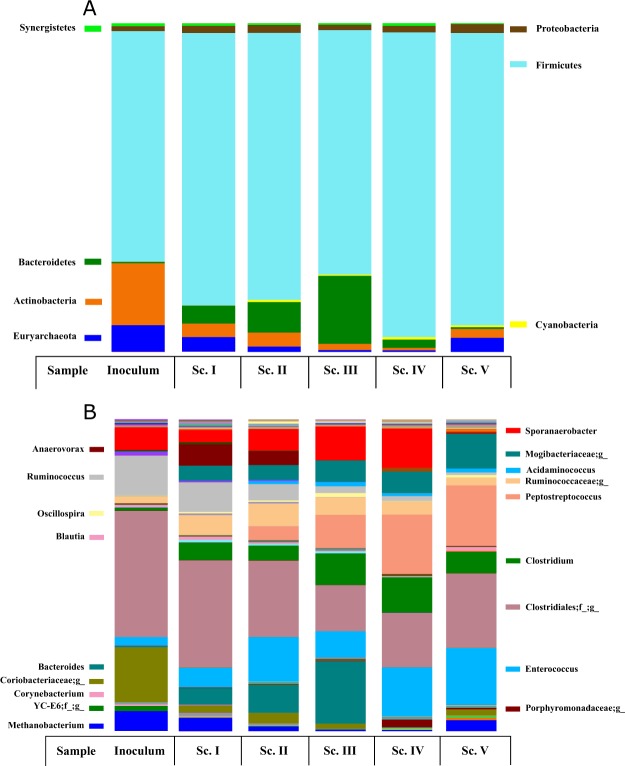
Main phyla (**A**) and genera (**B**) found during reactor operation.

The 16S rRNA gene analysis revealed that Firmicutes, Bacteroidetes and Actinobacteria were the most abundant phyla in the whole experimental period, further followed by Proteobacteria, Synergistetes and Euryarchaeota (Fig. 4A). The inoculum was dominated by Firmicutes phylum (70.2%), Actinobacteria (18.9%) and Euryarchaeota (8.1%). The high presence of bacteria belonging to Firmicutes phylum can be explained by the anaerobic sludge origin, which was an acidogenic anaerobic reactor (Section 2.1). Major contributors identified were species related with Clostridiales order (40.5%), other microorganism’s belonging to Coriobacteriaceae family (17.6%) as well as genera such as *Ruminococcus* (12.8%), *Sporanaerobacter* (7.2%) and *Methanobacterium* (6.4%). Overall, the community structure in the sludge was composed by microorganisms exhibiting hydrolytic and acidogenic activities^43^.

At phylum level, the progressive OLR increase influenced the microbial population dynamics. During Sc. I, the first applied OLR provoked an increase in relevance of Firmicutes phylum (82.9%) and Bacteroidetes (5.4%) with a concomitant decrease of Actinobacteria (4.1%) and Euryarchaeota (4.4%) (Fig. 3a). Sc. II and Sc. III were characterized by the progressive disappearance of Actinobacteria and Euryarchaeota and the slight increase of Bacteroidetes (up to 20.7% in Sc. III). At this point it is important to highlight that Sc. III coincided with the highest organic matter conversions into VFAs obtained (Table 1). Thus, the balance established between Bacteroidetes and Firmicutes relative abundance as well as the reduction of the methanogenic activity (Euryarchaeota phylum) might play a key role in maximizing VFAs production. DNA analysis from Sc. IV and especially Sc. V showed a gradual increase of Firmicutes and Euryarchaeota together with the disappearance of Bacteroidetes (Fig. 4A). These factors likely caused the drop of organic matter into VFAs conversion registered at the end of the experimental time (Sc. V, Table 1). The dominance of Bacteroidetes and Firmicutes in acidogenic fermentation from grass biomass acidification was previously reported at 37 °C and 55 °C, respectively^44^. In addition, low diversity is encountered in acidogenic reactors when compared to populations observed in AD processes devoted to biogas production. In fact, similar studies using microalgae as feedstock for methane generation revealed higher phylum diversity than in the study presented herein. Gonzalez-Fernandez *et al*. (2018) used *Chlorella sorokiniana* and *Scenedesmus* sp. for methane generation and obtained a diverse community characterized by the presence of different phyla such as Proteobacteria (46–51%), Firmicutes (20%), Bacteroidetes (2–6%) and Euryarchaeota (7–8%)^45^. This latter study and the one carried out by Greses and co-workers^46^ showed the low presence of Bacteroidetes in a process devoted for biogas production. Moreover, the relative abundances (%) in those studies between Bacteroidetes and Euryarchaeota were very different to those reported in the present investigation where Bacteroidetes stood out when methanogenic species were suppressed. This combination resulted in high VFAs productions. Following the same trend, Proteobacteria is another phylum which showed variation between acidogenic fermentation and AD for biogas production. Whereas in the present study this phylum showed values below 3% other studies devoted to biogas production reported values drastically different (46–51%)^45^. Moreover, the low COD removals percentages achieved along the experiment (Table 1) suggested that the syntrophic association among Proteobacteria and Euryarchaeota was weakened. Overall, it could be stated that the existing differences in terms of microbial population between an anaerobic community devoted to biogas production and the acidogenic inoculum presented herein indicated that the inoculum chosen was appropriate for VFAs maximization.

At genera level, operational conditions affected species differently. Whereas some of them gradually disappeared such as *Ruminococcus*, *Anaerovorax*, or microorganisms related with the Coriobacteriaceae family, others increased its relative abundance. In this sense, *Sporanaerobacter, Clostridium, Peptostreptococcus* and *Enterococcus* belonging to Firmicutes phylum gained importance along the experiment. More specifically, *Sporanaerobacter* has been identified in acidogenic reactors fed with microalgae biomass and has been pointed out to be responsible of metabolizing sugars, peptides and single amino acids into acetate^47^. *Clostridium* genus is involved in butyrate, acetic acid, lactic acid and ethanol production due to their ability to carry out mixed acid and alcohol fermentations^48^, explaining the butyric acid dominance (from 25.8% in Sc. I to 32.2% in Sc. V) in the VFAs profile as well as the high acetic acid productions (Fig. 2). *Peptostreptococcus* is associated with the presence of propionic and succinic acids in anaerobic digesters^49^. All of these species decreased their relative abundance during the last scenario contributing to the lower VFAs production attained (Fig. 1). In addition, the dominant genus found from Euryarchaeota phylum was *Methanobacterium*. The gradual decrease in terms of relative abundance of these genera (6.4% vs 0.5%) agreed with the concomitant drop of COD removals percentages encountered throughout the process (Table 1). Exception made for the last scenario, in which abundance levels raised once again (3.5%). This fact suggested that the hydrogenotrophic genus *Methanobacterium* was able to get adapted at the end of the experimental time. Hydrogenotrophic species are reported to be more resistant than acetoclastic methanogens to high VFAs and ammonium concentrations^50,51^. In fact acetic acid accumulated (Fig. 2) but no acetoclastic species were found along the evaluated scenarios. Thus, harsh operational conditions (low HRT and high OLR), causing the wash out of archaea species, as well as the high ammonium and VFAs levels detected most likely explained the acetoclastic inhibition and the low hydrogenotrophic methanogens presence^32^. The high tolerance of this latter species was demonstrated during Sc. IV and V, in which no VFAs enhancement was reported.

### VFAs applications, future perspective and challenges

VFAs are currently produced via the petro-chemical pathway. Nevertheless, their production through AD via the carboxylate platform is an attractive biotechnological technology to valorize organic residues in an environmentally friendly manner. Transition into a circular economy based on sustainable use of resources is, nowadays, a must. For this purpose, waste streams constitute a cost-effective raw material from which volatile fatty acids (VFAs) can be obtained through AD. AD has been mainly devoted to biogas production. Nevertheless, carboxylate production through this via requires further research.

This bioprocess does not require sterilization and hence, lower capital and operating costs compared to axenic cultures are involved. The economic value of the VFAs generally relies on the chain length. The market price increases from acetic (600$/ton) to butyric acids (2163$/ton)^52^. VFAs have been used for a wide variety of applications such as ester, alcohols production, food preservatives^2^. The possibility of elongating VFAs through the reverse ß oxidation pathway or single cell oils is being currently studied to give additional value to the products obtained^53,54^. In addition, these chemicals have gained importance in the biofuels field, namely biodiesel^55^ and biohydrogen^56^ production or electricity generation via microbial fuel cells^57^. Lastly, they can also be used for biopolymers production. However, distribution of these chemicals (VFAs profile) must be considered for some applications such as their use for polyhydroxyalkanoates production (PHAs). In this sense, a specific VFAs profile implies predictable PHAs characteristics. The prevalence of acids with even number of carbons promotes 3-hydroxybutyrate synthesis whereas 3-hydroxyvalerate is favoured by odd number VFAs^58^.

Despite of their wide potential, there are still barriers that require to be overcome in order to implement the carboxylate technology at industrial scale. For instance, the methanogenic step of the AD must be suppressed to favour VFAs accumulation in the digestate by using chemicals^59^ or operational strategies (low HRTs and high OLRs, as presented herein). Likewise, more research should be conducted to avoid acidogenic inhibition that may result from the high VFAs and ammonium concentration^37^. In this sense, efforts need to be directed to identify the key acidogens and to apply different process configurations and operational conditions to overcome inhibitory effects. For instance, when conversion yields are decreased at high OLR, increasing the HRT or bioaugment the anaerobic microbiome might be beneficial.

Some other technological key aspects rely on the VFA application. Whereas biogas is easily separated from the digestate, separation and purification steps might be required depending on the VFAs application. The product quality needed for a specific application will determine the separation method employed^60^. Hence, the proper choice of the separation method is of paramount importance for the process to be cost effective. In terms of the biology of the system, there are more topics deserving further investigation such as the production of targeted VFAs or the anaerobic microbiome response towards operational changes or potential perturbation that the system might suffer.

Overall, the carboxylate platform might become an efficient technology to produce value added-products from microalgae biomass. The flexibility of anaerobic digestion towards different organic feedstocks and the alternative products that can be attained further than biogas, makes this technology an important producer of valuable chemicals. Nevertheless, as aforementioned, more research is required to move from the most conventional product (biogas) to the new bio-based materials required in the chemical industry.

## Conclusions

VFAs production yields and microbial populations were affected by increasing OLRs. Butyric, acetic and propionic acids were the most abundant products (23–32%, 19–26%, 11–17%, respectively, out of the total VFAs). Organic matter conversion into VFAs was maximized at 9 and 12 g COD L^−1^ d^−1^ reaching VFAs productions up to 36.8 ± 2.1 g COD-VFA L^−1^ (conversion yields of 0.37 ± 0.02 COD-VFAs/COD_in_). During these stages a good balance between microbial populations (Bacteroidetes and Firmicutes) as well as low methanogenic presence was observed. However, the last scenario (OLR 15 g COD L^−1^ d^−1^) did not report an enhancement on VFAs productions. VFAs production was hampered at the acidogenic stage due to the combined effect of high ammonium, sodium and VFAs concentrations. The microbial follow up in this latter scenario revealed a reduction of Bacteroidetes phylum as well as the increase of methanogenic population. This microbial shift was found crucial in the lower organic matter conversions into VFAs obtained in Sc. V.

## Supplementary information

Supplementary information

## Data Availability

All data generated or analyzed during this study are included in this published article and Additional file 1.
