# Supplementary material for: Impact of Organic Loading Rate in Volatile Fatty Acids Production and Population Dynamics Using Microalgae Biomass as Substrate
Source: Sci Rep. 2019 Dec 5;9:18374. doi: 10.1038/s41598-019-54914-4 (PMC6895168; doi:10.1038/s41598-019-54914-4)
Supplement: Supplementary file 1 — Supplementary information [file 41598_2019_54914_MOESM1_ESM.pdf]

IMPACT OF ORGANIC LOADING RATE IN VOLATILE FATTY ACIDS PRODUCTION AND POPULATION DYNAMICS USING MICROALGAE BIOMASS AS SUBSTRATE

Jose Antonio Magdalena[1], Silvia Greses[1], Cristina González-Fernández[1*]

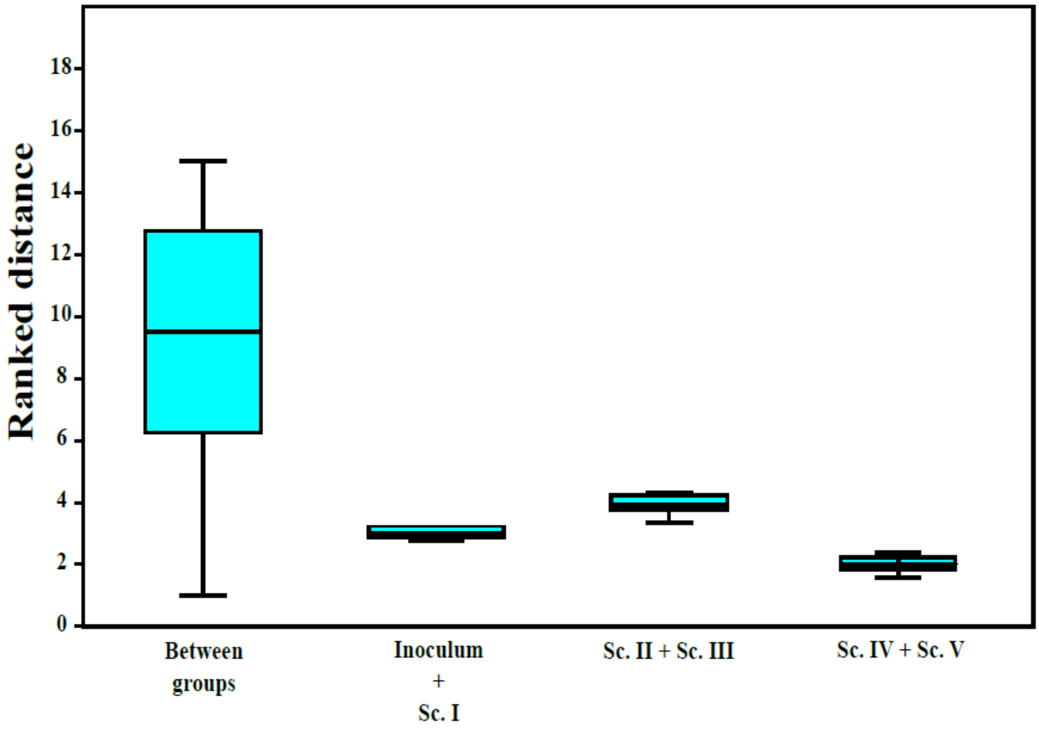

Figure S1. ANOSIM test carried out for the different scenarios

Table S1. R values of ANOSIM test:

	Inoculum +Sc. I	Sc. II + Sc. III	Sc. IV + Sc V
Inoculum +Sc. I	-	0.5	1
Sc. II + Sc. III	0.5	-	1
Sc. IV + Sc V	1	1	-
